# Ventricular arrhythmia burden in patients with implantable cardioverter defibrillator and remote patient monitoring during different time intervals of the COVID-19 pandemic

**DOI:** 10.1186/s40001-022-00867-w

**Published:** 2022-11-08

**Authors:** Christian Hauck, Andreas Schober, Alexander Schober, Sabine Fredersdorf, Ute Hubauer, Lars Maier, Andreas Keyser, Moritz Huttelmaier, Thomas Fischer, Carsten Jungbauer, Ekrem Ücer

**Affiliations:** 1 Department of Internal Medicine II, University Medical Center Regensburg, Franz-Josef-Strauß-Allee 11, 93053 Regensburg, Germany; 2 Department of Cardiothoracic Surgery, University Medical Center Regensburg, Franz-Josef-Strauß-Allee 11, 93053 Regensburg, Germany; 3 Department of Internal Medicine I, University of Wuerzburg, University Hospital, Oberdürrbacherstraße 6, 97080 Wuerzburg, Germany

**Keywords:** COVID-19, Implantable cardioverter defibrillator, Remote patient monitoring, Arrhythmia

## Abstract

**Purpose:**

The current study investigated whether the changes in patient care in times of the COVID-19 pandemic, especially the reduction of in-person visits, would result in a deterioration of the arrhythmic and clinical condition of patients with an implantable cardioverter defibrillator (ICD) and remote patient monitoring.

**Methods:**

Data were obtained from a local ICD registry. 140 patients who received ICD implantation at our department and had remote patient monitoring were included. The number of patients with ventricular arrhythmias, appropriate ICD therapy, the number of visits to our outpatient clinic and hospitalization due to acute coronary syndrome, stroke or heart failure were compared during three time intervals of the COVID-19 pandemic (first (LD1) and second (LD2) national lockdown in Germany and the time after the first lockdown (postLD1)) and a time interval 1 year before the pandemic began (preCOV). Each time interval was 49 days long.

**Results:**

Patients had significantly fewer visits to our outpatient clinic during LD1 (*n* = 13), postLD1 (*n* = 22) and LD2 (*n* = 23) compared to the time interval before the pandemic (*n* = 43, each *p* ≤ 0.05). The number of patients with sustained ventricular arrhythmias, appropriate ICD therapy and clinical events showed no significant difference during the time intervals of the COVID-19 pandemic and the time interval 1 year prior.

**Conclusions:**

The lockdown measures necessary to reduce the risk of infection during the COVID-19 pandemic, led to a reduction of in-person patient visits, but did not result in a deterioration of the arrhythmic and clinical condition of ICD patients with remote patient monitoring.

## Introduction

The first cases of COVID-19 infections were described in the Chinese city of Wuhan in late December 2019. The World Health Organization declared COVID-19 a global pandemic on March 12th, 2020 and with rising numbers of infections the first national lockdown in Germany was implemented on March 16th, 2020. During the pandemic, hospitals had to reduce in-person visits, especially in outpatient clinics, to reduce the risk of infections for healthcare workers and patients. Furthermore, clinics had to provide resources for clinical care of COVID-19 patients. Therefore, the Heart Rhythm Society (HRS) recommended to use remote monitoring for patients with cardiac implantable electronic devices (CIED) in most circumstances to reduce the need for non-urgent clinic visits [1]. An EHRA physician survey found a significant increase in the use of remote monitoring in patients with a pacemaker during the COVID-19 pandemic, but this effect was not seen in patients with an implantable cardioverter defibrillator (ICD) [2].

Patients with an ICD usually suffer from severe chronic cardiac conditions. ICD for primary prevention is especially recommended in patients with heart failure and reduced ejection fraction (HFrEF) [3, 4]. Secondary prevention indication for an ICD includes survival of sudden cardiac death (SCD) or sustained ventricular arrhythmias [5–8].

Regular visits to the outpatient clinic are important for ICD patients to control the device for episodes of arrhythmias, device therapy and technical functionality. Furthermore, changes in the patient’s clinical status could be recognized. Studies have shown that patient visits can be reduced safely with remote patient monitoring [9–11]. As all of these studies have been conducted in pre-pandemic times, it is not clear whether a sudden reduction of patient visits due to the lockdown measures caused by the COVID-19 pandemic has an influence on the outcome of ICD patients with remote patient monitoring.

## Methods

### Study group

The current study included 140 patients from the University Hospital Regensburg ICD registry (Res-IST), who received ICD implantation in our institution and had remote patient monitoring for diverse reasons. Baseline data regarding clinical, echocardiographic, device parameters, medication as well as medical history were registered. The University Hospital Regensburg ICD registry (Res-IST) was approved by the institutional ethics committee and was, therefore, performed according to the ethical standards laid down in the 1964 Declaration of Helsinki and its later amendments.

### Remote patient monitoring

Patients with electrical device problems or with recurrent arrhythmias were included in remote patient monitoring. For this study, we analyzed the remote patient monitoring data from the Boston Scientific Latitude and the Abbott Merlin remote patient monitoring. Regular automatic transmissions were received at least once per week. In case of an electrical device problem, an event of an arrhythmia or ICD therapy automatic transmissions were received as soon as the patient was in contact with the remote monitoring device. All episodes of arrhythmias detected by the ICD device and all ICD therapies could be seen in the database. The IEGM of each episode was evaluated by a physician.

### Time intervals

We compared 4 time intervals and each time interval was 49 days long. 3 time intervals were during the COVID-19 pandemic. The time interval from March 16th, 2020 to May 03rd, 2020 was during the first national lockdown in Germany due to the pandemic (LD1). The time interval from May 04th, 2020 to June 21st, 2020 was the period following the first lockdown, with a stepwise reopening of schools, businesses, and restaurants (postLD1). The time interval from December 16th, 2020 to February 02nd, 2021 was during the second lockdown due to the second wave of COVID-19 infections in Germany (LD2). We compared these 3 intervals to the period from March 16th, 2019 to May 03rd, 2019, 1 year before the COVID-19 pandemic caused the first national lockdown (preCOV).

### Outcomes

The purpose of this study was to investigate the effect of the COVID-19 pandemic on the clinical condition of ICD patients with remote patient monitoring. Therefore, we analyzed the number of patients with sustained ventricular arrhythmias, which were defined as ventricular tachycardia or ventricular fibrillation lasting longer than 30 s or being treated adequately by anti-tachycardia pacing (ATP) or ICD shock. The number of non-sustained ventricular arrhythmias and all ventricular arrhythmias were also investigated. All appropriate ICD therapies were evaluated. Appropriate ICD therapy was defined as ATP and/or ICD shock due to ventricular arrhythmias. Furthermore, the number of visits to our ICD outpatient clinic during the different time intervals was analyzed. Finally, the number of patients with a hospitalization in our institution due to myocardial infarction, stroke or heart failure was evaluated. In August 2022 all patients that took part in the current study were contacted by phone and mail to obtain further information about infections with COVID-19 and treatments with hydroxychloroquine and azithromycin during any of the time intervals of the study.

### Statistical analysis

Categorical variables are presented as counts and percentages (%). Continuous variables are presented as mean ± standard deviation (SD). Mc Nemar-Test was used for categorical variables and Wilcoxon signed-rank test was used for means of continuous variables to test statistical significance. The selected endpoints were compared between 4 different time intervals. A *p* value ≤ 0.05 was considered statistically significant. For statistical analysis, IBM Statistics SPSS Version 25 was used.

## Results

### Baseline characteristics

The baseline characteristics of all 140 patients are presented in Table 1. Patients had a mean age of 58.9 years and the majority (82.9%) were male. The mean LVEF was 40% ± 14%. 41.4% of all patients received an ICD for primary prevention. 30.0% of all patients received an ICD because of ventricular fibrillation and 27.9% because of ventricular tachycardia. Ischemic heart disease was present in 47.1% and 7.1% had a history of stroke. 20.0% suffered from chronic kidney diseases (CKD).

**Table 1:** Baseline characteristics

	*n* = 140
Age	58.9 (± 17.2)
Men	116 (82.9%)
LVEF	40% (± 14)
Primary prevention	58 (41.4%)
Secondary prevention	82 (58.6%)
ICD indication VF	42 (30.0%)
ICD indication VT	39 (27.9%)
ICD indication others^a^	1 (0.7%)
Single-chamber ICD	78 (55.7%)
Dual-chamber ICD	20 (14.3%)
S-ICD	14 (10.0%)
CRT-D	28 (20.0%)
IHD	66 (47.1%)
DCM	36 (25.7%)
Others^b^	41 (29.3%)
History of MI	43 (30.7%)
Diabetes	29 (20.7%)
Hypertension	78 (55.7%)
Obesity (BMI ≥ 30)	37 (26.4%)
Hyperlipidemia	64 (45.7%)
History of stroke	10 (7.1%)
PAD	7 (5.0%)
Carotis stenosis	3 (2.1%)
CKD	28 (20.0%)
COPD	6 (4.3%)
ACE/AT1/ARNI	101 (72.1%)
Beta blocker	118 (84.3%)
Spironolactone	82 (58.6%)
Diuretics	87 (62.1%)
Amiodarone/Sotalol	10 (7.1%)
Digitalis	9 (6.4%)

*LVEF* left ventricular ejection fraction, *ICD* implantable cardioverter–defibrillator, *VF* ventricular fibrillation, *VT* ventricular tachycardia, *S-ICD* subcutaneous implantable cardioverter–defibrillator, *CRT* cardiac resynchronization therapy, *IHD* ischemic heart disease, *DCM* dilated cardiomyopathy, *BMI* body mass index, *MI* myocardial infarction, *PAD *peripheral artery disease, *CKD* chronic kidney disease, *COPD* chronic obstructive pulmonary disease

ICD indication others^a^: patients with an out of hospital cardiac arrest (OHCA) and high probability of a primary rhythmogenic cause, though first documented rhythm was asystole, PEA or sinus rhythm

Others^b^: primary VF: 9 (6.4%), long-QT: 7 (5.0%), hypertrophic cardiomyopathy: 7 (5.0%), myocarditis: 6 (4.3%), Tako Tsubo cardiomyopathy: 3 (2.1%), secondary cardiomyopathy: 3 (2.1%), arrhythmogenic right ventricular dysplasia: 1 (0.7%), Brugada: 2 (1.4%), non-compaction cardiomyopathy: 1 (0.7%), muscular dystrophy: 1 (0.7%), short-QT: 1 (0.7%)

### Visits to the ICD outpatient clinic

**Fig. 1:**
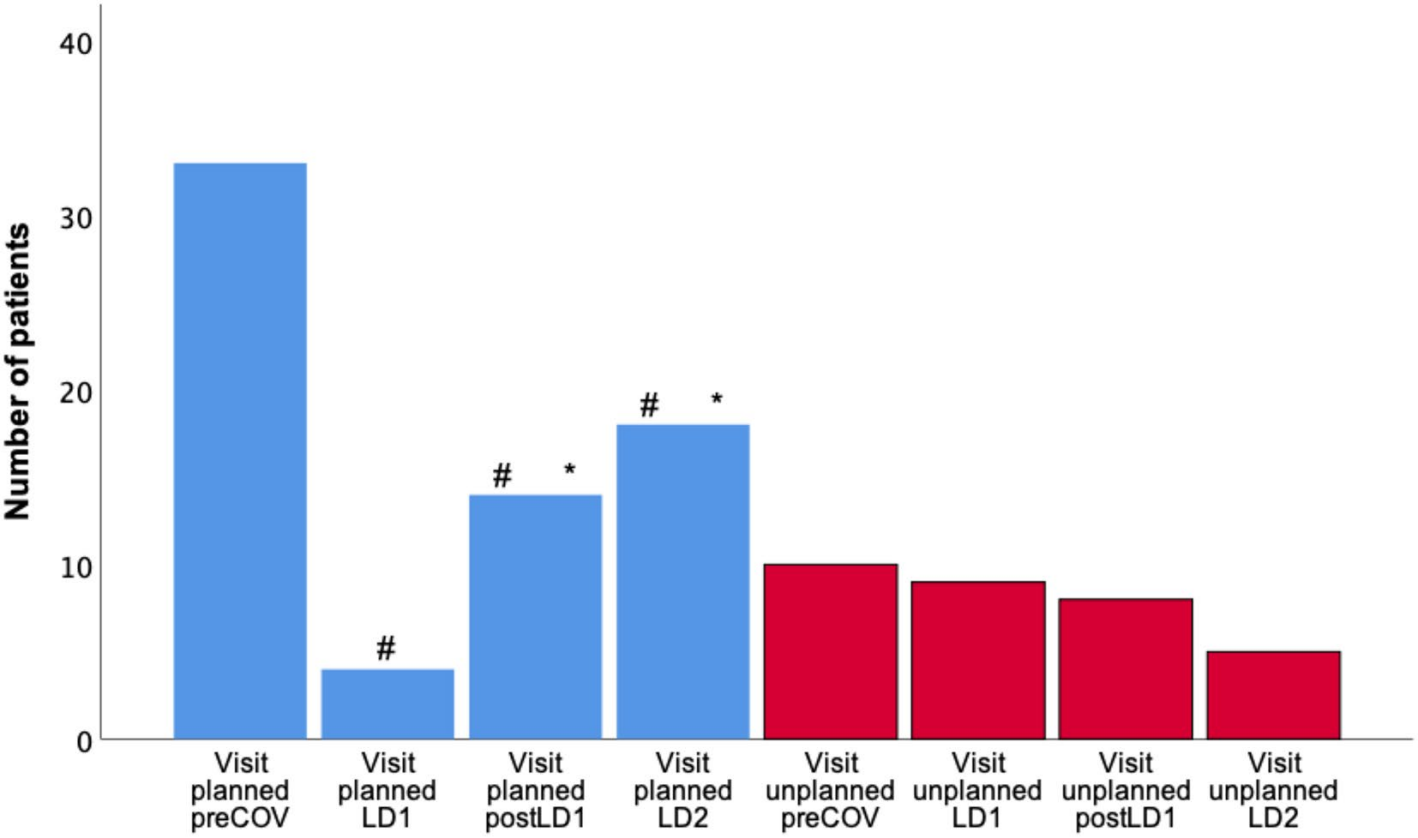
Patients with visits to the ICD outpatient clinic. #Significant differences compared to preCOV. *Significant differences compared to LD1

Analyzing the number of visits to the ICD outpatient clinic of our institution, 43 patients (30.7%) had a visit during preCOV, 13 patients (9.3%) during LD1, 22 patients (15.7%) during postLD1 and 23 patients (16.4%) during LD2. Patients had significantly fewer visits during LD1 (*p* < 0.001), post LD1 (*p* = 0.002) and LD2 (*p* = 0.006) compared to preCOV. Comparing the three time intervals during the pandemic, no significant difference could be found (each *p* = n.s.) (Fig. 1).

There were significantly fewer planned visits to the ICD outpatient clinic during all three time intervals of the pandemic compared to preCOV (preCOV *n* = 36; LD1 *n* = 4, *p* < 0.001; postLD1 *n* = 14, *p* < 0.001; LD2 *n* = 19, *p* = 0.007). Furthermore, duringLD1 significantly fewer planned visits took place compared to every other time interval (preCOV *n* = 36, *p* < 0.001; postLD1 *n* = 14, *p* = 0.013; LD2 *n* = 19, *p* < 0.001).

Regarding unplanned visits, no significant difference comparing preCOV (*n* = 16) to LD1 (*n* = 10) and postLD1 (*n* = 10) could be seen (each *p* = n.s.). There were significantly more unplanned visits during preCOV comparing to LD2 (*n* = 6) (*p* = 0.013).

The programming of the ICD was significantly more often changed during preCOV compared to LD1, post LD1 and LD2 (Table 2), whereas no difference was found between the three time intervals during the pandemic (each *p* = n.s.).

**Table 2:** Changes in ICD programming

Time periods	Number of patients	Compared to preCOV
preCOV	15	
LD1	5	*p* = 0.021
postLD1	2	*p* = 0.001
LD2	1	*p* = 0.001

Differences between any time interval during the COVID-19 pandemic were not significant

### Ventricular arrhythmias

**Fig. 2:**
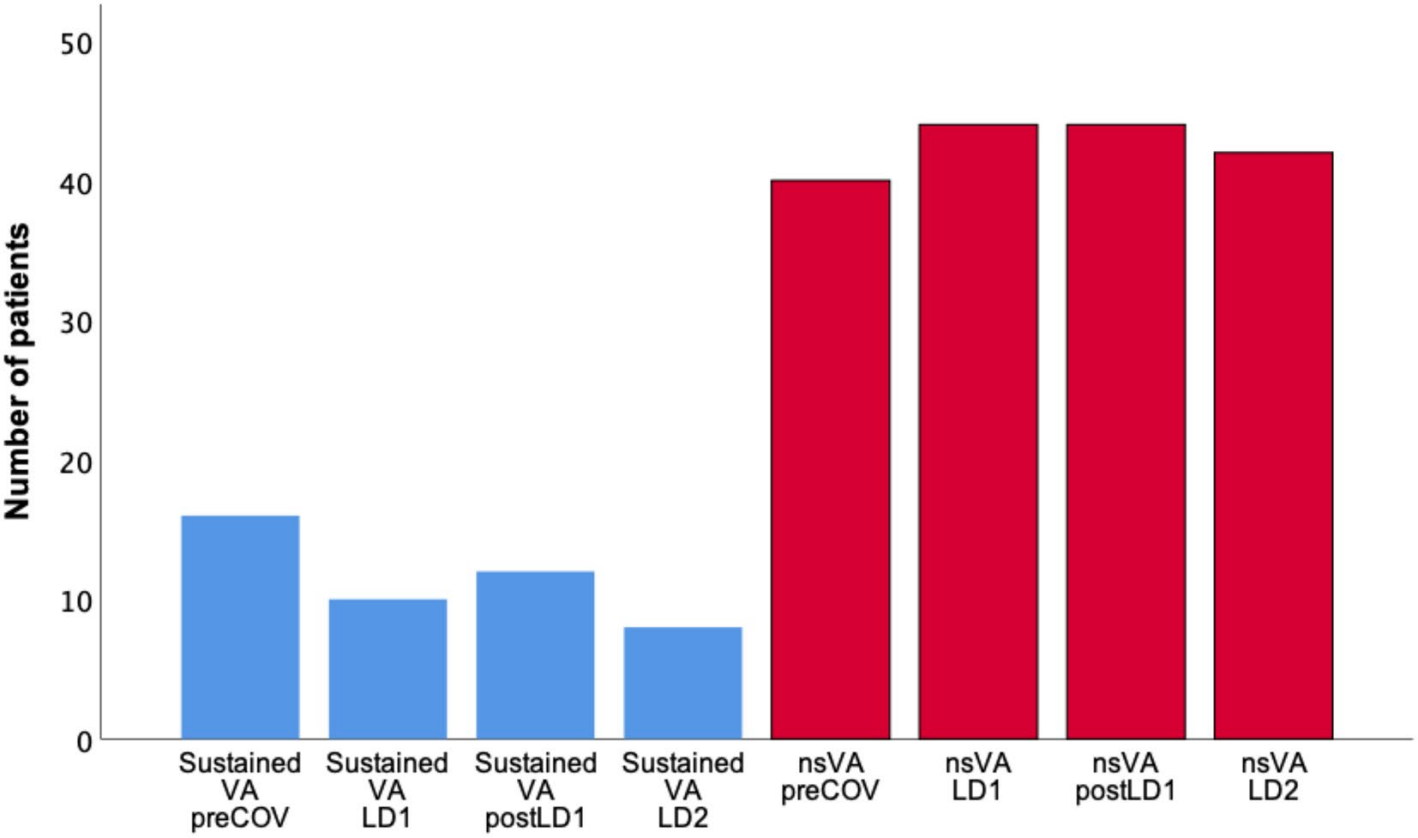
Patients with ventricular arrhythmias

16 patients (11.4%) had episodes of sustained ventricular arrhythmias during the preCOV interval. During LD1 10 patients (7.1%) suffered from sustained ventricular arrhythmias. During postLD1 12 patients (8.6%) and during LD2 8 patients (5.7%) had sustained ventricular arrhythmias. There was no significant difference between any time interval (each *p* = n.s.) (Fig. 2; Table 3).

**Table 3:** Number of patients with ventricular arrhythmias

Time periods	VA	Sustained VT	VF	Non-sustained VT
preCOV	44	15	2	40
LD1	45	9	1	44
postLD1	45	9	3	44
LD2	44	8	0	42

Differences between any time interval were not significant

Regarding the number of episodes with ventricular arrhythmias also no significant difference could be seen between the time intervals (each *p* = n.s.) (Table 4).

**Table 4:** Number of ventricular arrhythmias

Time periods	VA	Sustained VT	VF	Non-sustained VT
preCOV	958	98	2	859
LD1	849	99	1	749
postLD1	899	111	3	959
LD2	866	114	0	752

Differences between any time interval were not significant

There were no significant differences regarding the different types of sustained ventricular arrhythmias (sustained ventricular tachycardia, ventricular fibrillation) (each *p* = n.s.). The number of patients with sustained ventricular tachycardia was 15 (10.7%) during preCOV, 9 (6.4%) during LD1, 9 (6.4%) during postLD1 and 8 (5.7%) during LD2 (Table 3). In addition, the number of episodes of sustained VT did not differ between the time intervals (each *p* = n.s.) (Table 4).

The number of patients with ventricular fibrillation and mean number of episodes with ventricular fibrillation was low during all time intervals with no significant difference (each *p* = n.s.) (Tables 3, 4).

Non-sustained ventricular arrhythmias were seen in 40 patients (28.6%) during preCOV, in 44 patients (31.4%) during LD1, in 44 patients (31.4%) during postLD1 and in 42 patients (30.0%) during LD2. Regarding the number of patients with non-sustained ventricular arrhythmias and the number of episodes with non-sustained ventricular arrhythmias, no significant difference could be found (each *p* = n.s.) (Fig. 2; Tables 3, 4).

Finally, looking at all ventricular arrhythmias (sustained ventricular tachycardia, ventricular fibrillation, non-sustained ventricular tachycardia) no difference was seen regarding the number of patients suffering from any episode of ventricular arrhythmias and the number of episodes of ventricular arrhythmias during any time interval (each *p* = n.s.) (Tables 3, 4).

### ICD therapy

**Fig. 3:**
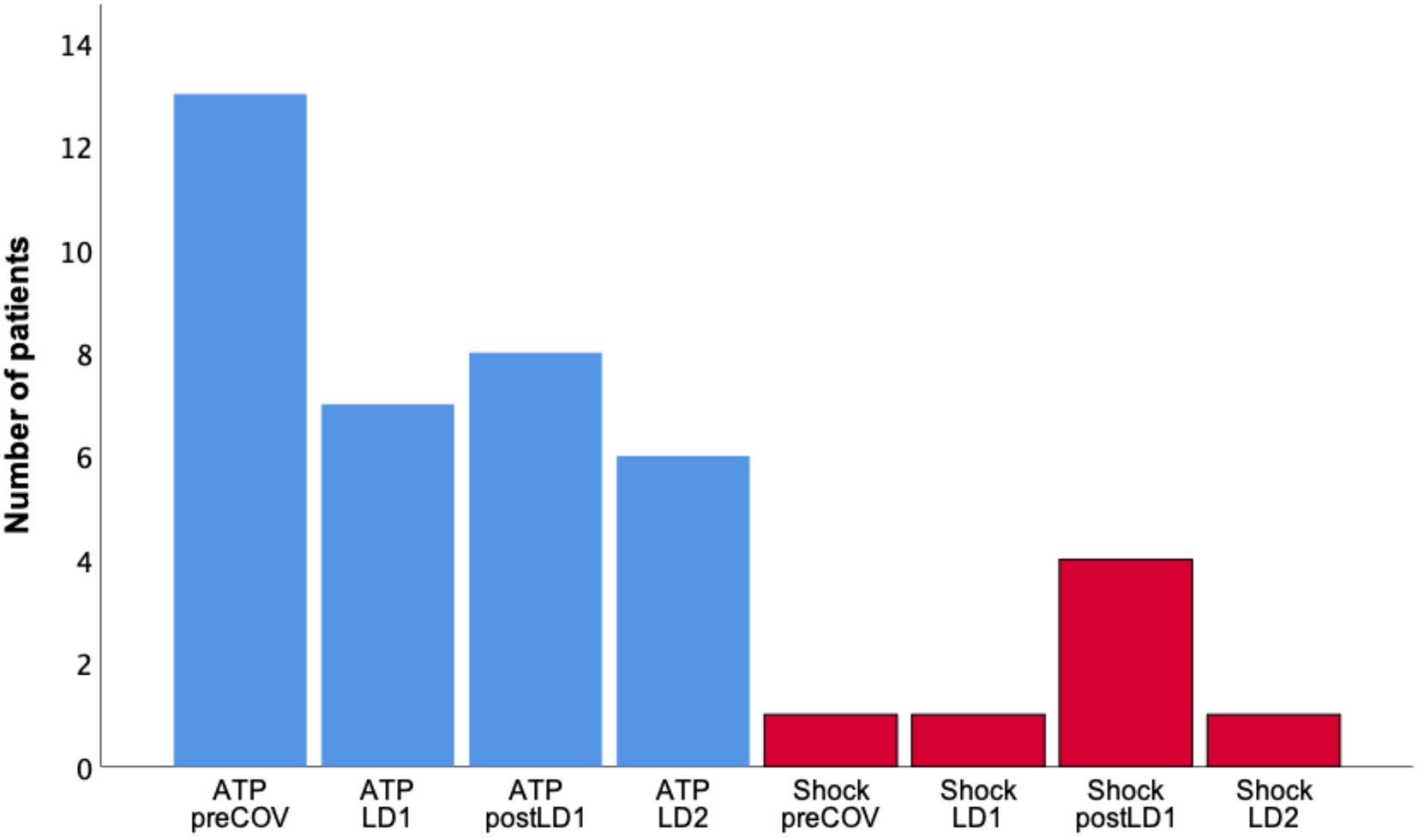
Patients with ICD therapy

14 patients (10.0%) during preCOV, 8 patients (5.7%) during LD1, 11 patients (7.9%) during postLD1 and 6 patients (4.3%) during LD2 had adequate ICD therapy (ATP and/or ICD shock) to treat ventricular arrhythmias. Regarding the number of patients with ICD therapy and the number of episodes with ICD therapy, no significant difference was seen (each *p* = n.s.) (Fig. 3).

With regard to the different types of ICD therapy (ATP and ICD shock) we could find no significant difference during the time intervals (each *p* = n.s.) (Fig. 3).

### Hospitalization

During every time interval only 1 patient was hospitalized because of myocardial infarction, stroke or heart failure, so that no significant difference regarding this clinical end point could be seen (each *p* = n.s.).

### COVID-19 infections

131 patients of the study group could be contacted to get information about COVID-19 infections and the treatment with hydroxychloroquine and azithromycin. No information could be obtained from 7 patients, who died since the end of the study, and no family members of these patients could be contacted. Furthermore, 2 patients, who don’t live in Germany any longer, could not be reached by phone and mail.

One patient suffered from an infection with COVID-19 during LD1. The patient was not hospitalized and no medication with hydroxychloroquine and azithromycin was given. No patient had an infection with COVID-19 during postLD1 and LD2.

## Discussion

In our single center registry study, no significant difference could be seen regarding the occurrence of ventricular arrhythmias and ICD therapies in ICD patients with remote patient monitoring comparing two time intervals during and one time interval after the first national lockdown in Germany due to the COVID-19 pandemic and a time interval 1 year prior to the COVID-19 pandemic. There was also no difference in respect to clinical events, which were very low during each time interval. During the COVID-19 pandemic, there were significantly fewer planned visits to our ICD outpatient clinic, which did not result in a worsening of the rhythmologic or clinical condition of ICD patients with remote patient monitoring.

### In-person-visits to the ICD outpatient clinic

When the COVID-19 pandemic reached Europe and the United States in March 2020 in-person-visits to outpatient providers declined. In our study, in-person-visits to our outpatient ICD clinic were almost halved during the first national lockdown in Germany compared to the same time interval 1 year before. Planned visits even declined by 89%, whereas unplanned visits showed no difference. There were still significantly fewer planned visits during the 7 weeks after the first lockdown and during the second lockdown. On one hand hospitals were still trying to reduce in-person visits, on the other hand patients themselves also were trying to avoid visits to the hospital to avoid a possible risk of infection. In an analysis of US insurance data from January 2020 to June 2020 a decline of in-person-visits to outpatient providers by 30.0% could be seen [12]. Another study that presented data from the US Outpatient Influenza-like Illness Surveillance Network saw a 70% decline of outpatient visits during April 5th to April 11th, 2020 compared to the same period 1 year before [13]. In the current study, the strong decline in planned visits was due to the fact that only patients with remote patient monitoring were investigated.

### Ventricular arrhythmias and ICD therapy

The current study showed no significant difference between any time interval regarding the occurrence of ventricular arrhythmias and ICD therapies. Similar results were found by a recent study by Sassone et al. [14] The authors compared a 10-week long time interval of the first lockdown in Italy to the time interval before the lockdown began and the corresponding time interval 1 year earlier. No differences in the occurrence of arrhythmias and ICD therapies were found in this study. In difference to the current study, the investigators included all patients with an ICD regardless of whether the patients had access to remote patient monitoring. Furthermore, the time interval after the lockdown and the time interval during the second lockdown were not investigated. Another current study compared ICD patients in whom the regular in-person-visit was replaced by a remote patient monitoring interrogation to patients who recently had their regular in-person-visit. Regarding the occurrence of arrhythmic events, no significant difference between the two groups could be found [15]. The study design of this study was completely different compared to the current study. The authors compared an intervention group of 131 patients to a control group of 198 patients during 1 month of the pandemic, whereas the present study investigated the differences of one group of 140 ICD patients with remote patient monitoring during 4 different 7-week long-time intervals during and before the pandemic. Therefore, it was conducted over a longer period and also investigated the effect of different time intervals of the pandemic with different lockdown measures on the rhythmologic and clinical situation of ICD patients with remote patient monitoring. Several studies have shown that an infection with COVID-19 could be a potential trigger for arrhythmias [16–18]. Although atrial arrhythmias were detected more frequently in patients hospitalized with COVID-19, ventricular arrhythmias may especially play a role in patients with preexisting cardiac conditions, such as ischemic heart disease [17, 19]. A case report even described a patient with an electrical storm, which was potentially triggered by an infection with COVID-19 [20]. Several mechanisms have been discussed to explain the high incidence of arrhythmias in patients hospitalized with COVID-19. Cytokine storm, mediated by an imbalanced response among subtypes of T-helper cells and hypoxia-induced intracellular calcium overload leading to early afterdepolarization can contribute to trigger ventricular arrhythmias [19–22]. Myocardial injury, myocarditis, direct viral invasion or the use of QT prolonging drugs such as hydroxychloroquine and azithromycin may also lead to the occurrence of ventricular arrhythmias [18, 23–26]. On the other hand, a study by Gasperetti et. al that described ECG modifications and arrhythmic events in COVID-19 patients undergoing hydroxychloroquine therapy found only modest QTc prolongation, a low ventricular arrhythmia rate of 1.1% and no arrhythmic-related deaths [27]. In the current study, only one patient had an infection with COVID-19 during LD1. This patient was not hospitalized or treated with hydroxychloroquine or azithromycin. No further infections occurred during the other investigated time intervals of the pandemic. Therefore, we can exclude that the beforementioned factors had a relevant influence on the number of ventricular arrhythmias in the present study. The reason for this finding may be that the lockdown measures during the first year of the pandemic and the behavior of chronically ill patients with an ICD, who may have been very careful to avoid an infection, could have led to a very low incidence of COVID-19 infections in the study group.

### Clinical events

It's still a matter of debate, whether the decline in patient visits results in a worsening of chronic illnesses, such as chronic heart failure or coronary artery disease. Clinical events were low during every time interval, and no difference could be seen to the time interval 1 year prior to the pandemic. Only one patient died between the time intervals postLD1 and LD2. Comparable to the present findings, the aforementioned study by Sassone et al. also found no increase in arrhythmic death during the first national lockdown in Italy [14].

Unplanned visits to our ICD outpatient clinic, including problems detected by remote patient monitoring, hospitalized patients and patients presenting in the emergency room showed no significant difference during the different time intervals of the pandemic.

As the investigated time intervals were only 7 weeks long, one can still speculate whether changes in patients’ behavior during the pandemic, such as reduction of physical activity and social distancing, might have any influence on the medical condition of chronically ill patients over a longer time period.

In contrast to the present findings, a nationwide survey in Italy found a reduction in admissions for acute myocardial infarction of 48.4% comparing a week in March 2020 to the equivalent week in 2019, but this resulted in a substantially increased STEMI fatality rate [28]. As this was a nationwide survey, which looked at the outcome of patients with acute myocardial infarction, the results cannot be compared to our single center study with a completely different group of patients. ICD patients often have a long history of chronic heart failure or coronary artery disease and might recognize an increase of symptoms early enough to contact their medical provider and avoid further worsening.

A rise of the arrhythmic burden could be an early indicator for a worsening of chronic cardiac conditions. We didn't see any difference regarding the number of patients with ventricular arrhythmias during any of the three time intervals of the pandemic and the interval before the pandemic. We can just speculate what the possible reasons might be, but the benefit of remote patient monitoring, which already has been described in pre-pandemic times, might also play an important role during the pandemic. The ALTITUDE Survival Study found a 50% reduction of mortality in patients with ICD and CRT-D and remote patient monitoring compared to usual care patients without remote patient monitoring [29]. Other studies have shown that remote patient monitoring enables the physician to see an episode of an arrhythmia faster and to react for example with further clinical examinations or adjustment of the medical therapy [9, 10, 30, 31]. Furthermore, patients who accept remote patient monitoring might be more compliant and more aware of their general health status and might seek a medical consultant early enough to avoid a further deterioration of their chronic condition. In sum, the close surveillance of the rhythmologic situation and the good compliance of ICD patients with remote patient monitoring could be possible reasons, why these patients are less negatively affected by a reduction of planned in-patient-visits to their medical provider.

The TRUST trial found that in a group of patients with an ICD and remote patient monitoring, the in-clinic and hospital visits could be reduced by 45% compared to the conventional care group. Despite fewer hospital visits, the detection of device specific events was advanced by 30 days and no difference in clinical adverse events could be seen [9]. Although the TRUST trial was conducted before the pandemic, the findings are very similar to the current study. Only one patient died between postLD1 and LD2, which may show that outpatient clinic visits for device interrogation can be reduced safely for ICD patients with remote patient monitoring during times of the pandemic without an increase of mortality and other clinically relevant events. Finally, the reduction of in-person-visits is an important aspect during a global pandemic regarding the reduction of potentially contagious situations for the patient and medical staff.

### Limitations

As we only included patients with remote patient monitoring and had no control group of ICD patients without remote patient monitoring, no statement can be made about the safety and outcome of ICD patients in general. Each investigated time interval was 7 weeks long due to the length of the first national lockdown in Germany. Therefore, we cannot say, whether a longer duration of lockdown measures would have caused other results. The retrospective non-randomized design makes a selection bias possible, as patients with remote patient monitoring are potentially more compliant and seek medical treatment early enough to avoid clinically relevant endpoints. Furthermore, we just registered hospitalizations in our institution. Therefore, we do not know whether patients were hospitalized in external hospitals. Because of the study design, we don’t have information about the number of COVID-19 infections in the study cohort during the different time intervals of the study. Finally, this was a single center study, and we do not know if our results can be generalized to other medical centers that might have other strategies for selecting patients eligible for remote patient monitoring.

## Conclusions

In this single center registry study, we found that in ICD patients with remote patient monitoring no rise of ventricular arrhythmias and clinical events could be seen during the COVID-19 pandemic. There were significantly fewer planned visits to our ICD outpatient clinic during the pandemic. In sum, the lockdown measures necessary to reduce the risk of infection during the pandemic, led to a reduction of direct patient contact but did not result in a worsening of the clinical condition of ICD patients with remote patient monitoring.

## Data Availability

Available from the corresponding author upon request.
